# Developmental Genetic Mechanisms of C_4_ Syndrome Based on Transcriptome Analysis of C_3_ Cotyledons and C_4_ Assimilating Shoots in *Haloxylon ammodendron*

**DOI:** 10.1371/journal.pone.0117175

**Published:** 2015-02-02

**Authors:** Yuanyuan Li, Xiuling Ma, Jialei Zhao, Jiajia Xu, Junfeng Shi, Xin-Guang Zhu, Yanxiu Zhao, Hui Zhang

**Affiliations:** 1 Key Laboratory of Systems Biology, Shandong Normal University, Jinan, Shandong, China; 2 Key Laboratory of Plant Stress Research, Shandong Normal University, Jinan, Shandong, China; 3 Key Laboratory of Computational Biology, CAS-MPG Partner Institute for Computational Biology, Shanghai Institutes for Biological Sciences, Chinese Academy of Sciences, Shanghai, China; Institute of Genetics and Developmental Biology, Chinese Academy of Sciences, CHINA

## Abstract

It is believed that transferring the C_4_ engine into C_3_ crops will greatly increase the yields of major C_3_ crops. Many efforts have been made since the 1960s, but relatively little success has been achieved because C_4_plant traits, referred to collectively as C_4_ syndrome, are very complex, and little is known about the genetic mechanisms involved. Unfortunately, there exists no ideal genetic model system to study C_4_ syndrome. It was previously reported that the *Haloxylon* species have different photosynthetic pathways in different photosynthetic organs, cotyledons and assimilating shoots. Here, we took advantage of the developmental switch from the C_3_ to the C_4_ pathway to study the genetic mechanisms behind this natural transition. We compared the transcriptomes of cotyledons and assimilating shoots using mRNA-Seq to gain insight into the molecular and cellular events associated with C_4_ syndrome. A total of 2959 differentially expressed genes [FDR≤0.001 and abs (|log_2_(Fold change)|≥1)] were identified, revealing that the transcriptomes of cotyledons and assimilating shoots are considerably different. We further identified a set of putative regulators of C_4_ syndrome. This study expands our understanding of the development of C_4_ syndrome and provides a new model system for future studies on the C_3_-to- C_4_ switch mechanism.

## Introduction

Photosynthetic CO_2_ fixation is a fundamental life process involving the conversion of solar energy into chemical energy that can be later released to fuel the activity of an organism. The ancestral photosynthetic CO_2_-fixation process is C_3_ photosynthesis. The first organic product of CO_2_ fixation is a three-carbon compound. C_3_ photosynthesis and its key enzyme Rubisco (ribulose-1,5-bisphosphate carboxylase/oxygenase) evolved early in the history of life, when there was no oxygen in the atmosphere and atmospheric CO_2_ levels were significantly high. As atmospheric CO_2_ dropped and O_2_ accumulated, Rubisco inefficiency began to limit C_3_ photosynthesis because the active site of Rubisco does not completely discriminate between CO_2_ and O_2_, leading to the catalysis of two competitive reactions: photosynthetic CO_2_ assimilation and photorespiratory CO_2_ loss, especially under hot, dry and/or saline conditions that enhance photorespiration. Approximately 30 million years ago, an abrupt drop in atmospheric [CO_2_] further reduced the efficiency of C_3_ photosynthesis and triggered the evolution of C_4_ photosynthesis, in which CO_2_ is initially fixed into a four-carbon compound and concentrated around Rubisco [1–6]. C_4_ photosynthesis has evolved >60 times and occurs in approximately 7500 species of flowering plants [7–9]. The transition from C_3_ to C_4_ plants was the green revolution of nature. Although C_4_ plants comprise only 3% of land plant species, they account for some 25% of global terrestrial carbon fixation [3,4,7,10,11].

C_4_ photosynthesis is a complex trait that combines biochemical, physiological and anatomical characteristics, the so-called C_4_ syndrome [12,13]. Other than four single-celled C_4_ lineages, the vast majority of C_4_ plants possess Kranz anatomy and C_4_ syndrome [8,9], indicative of convergent evolution [7]. Traditional biochemical and modern molecular biological studies showed that all of the proteins required for the core C_4_ cycle are present in C_3_ plants [14]. Based on this evidence, it is hypothesized that no significant genetic changes are required for the transition from C_3_ to C_4_ photosynthesis [15,16]. The first attempt at introducing C_4_ photosynthesis into C_3_ plants was made in *Atriplex* through conventional interspecific hybridization of photosynthetic types to reduce photorespiration and increase photosynthetic capacity [17–22]. The results indicated that F1 hybrids of *Atriplex rosea* (C_4_, NAD-ME type) × *Atriplex triangularis* (C_3_) were more similar to their C_3_ parents in physiology and failed to form well-developed Kranz anatomy. Similar hybridization studies were conducted in the genera *Flaveria*, *Panicum*, *Moricandia*, and *Brassica*, but none proved fruitful at converting C_3_ species into functional C_4_ types [23]. Due to infertility, few hybrids have been developed beyond the F1 generation. In the few advanced generations studied in *Atriplex* and *Flaveria* hybrids, correlations among photosynthetic traits were low, indicating that C_4_ photosynthesis is a combination of independent biochemical, physiological and anatomical characteristics [23]. Recently, a progress report was released on oat-maize addition lines showing that the addition of individual maize chromosomes to the C_3_ species oat caused increases in vein density but did not confer functional C_4_ photosynthesis [24]. Therefore, introducing a fully developed C_4_ photosynthesis pathway into C_3_ plants through interspecific hybridization or even genetic engineering is far more practical than previously thought [25].

In nature, there exist examples of switches from a C_3_ pathway to a two-celled C_4_ pathway triggered by external or internal signals. The former example includes the freshwater amphibious leafless sedge *Eleocharis vivipara*, which can switch from a C_3_ pathway to a C_4_ pathway with Kranz anatomy after induction by environmental changes and exogenous application of abscisic acid (ABA) [26–28]. The latter examples were reported in *Haloxylon* and *Salsola* species that have different photosynthetic pathways in different photosynthetic organs [29,30]. *Haloxylon aphyllum* and *H*. *persicum* of Chenopodiaceae have C_3_ photosynthesis in cotyledons and C_4_ photosynthesis in assimilating shoots (the main photosynthetic organs) with a typical Salsoloid-type Kranz anatomy [29]; the same phenomenon has been observed in *Salsola gemmascens* of the genus *Salsola* (Chenopodiaceae), in which cotyledons exhibit C_3_-type photosynthesis, while leaves perform NAD-malic enzyme (NAD-ME) C_4_-type photosynthesis with a Salsoloid-type Kranz anatomy [30]. However, this manner of C_3_-to-C_4_ switches has not received much attention, and there were no follow-up studies after their discovery. Investigations into the developmental genetic mechanisms controlling the different photosynthetic types in cotyledons and leaves or other photosynthetic organs will illuminate the genetic regulatory network of C_4_ syndrome.

With the development of new technologies, more studies have begun to analyze C_4_ syndrome at the systems biology level. To understand C_4_ formation, mesophyll cells and bundle sheath cells in the leaf blade of maize were used as a model system for C_4_ differentiation. The cells were separated and analyzed for transcriptional changes by microarray analysis and next-generation sequencing [31–33]; 21% of genes were differentially expressed between mesophyll cells and bundle sheath cells [32]. Recently, John et al. sequenced RNA isolated from the mesophyll cells and bundle sheath cells of *Setaria viridis* and found the significant convergence of cell-specific gene expression in *S*. *viridis* and maize [34]. Such studies deepen our understanding of C_4_ syndrome. In 2011, two research groups used mRNA-Seq analysis of closely related C_3_ and C_4_ species for which gene expression is altered, and these groups identified genes associated with the C_4_ pathway [35,36]. Up to 603 and 3582 transcripts differed in abundance between C_3_ and C_4_ leaves in the genera *Cleome* and *Flaveria*, respectively. While these two experiments were designed to identify C_4_-related transcriptomic gene expression changes, it is difficult to tell if the observed variation in transcript abundance was associated with differences between the species or C_4_ photosynthesis. However, the comparative transcriptomics of C_3_ cotyledons and C_4_ assimilating shoots in this study will identify more C_4_-specific genes than before because there is no genetic variation between these different species; more importantly, the natural developmental C_3_-to-C_4_ transition may give clues concerning its master switch.

## Materials and Methods

### Plant Growth and Harvesting

Seeds of *Haloxylon ammodendron* were provided by the Turpan Eremophyte Botanic Garden, Chinese Academy of Sciences in Turpan, Xinjiang, China (http://english.egi.cas.cn/rs/sr/tdbg/). The seeds were incubated and germinated on moist filter paper in Petri dishes. After germination, seedlings were planted in sand in a greenhouse maintained at 30/20°C day/night, 70% relative humidity and a photoperiod of 12 h light/12 h dark under a light intensity of 1000 µE m^–2^ s^–1^. Cotyledons were fully expanded after 2 days growing in sand and sampled for all analyses. Assimilating shoots were collected from plants at 10 days of age. For mRNA-Seq, samples were taken from 10–15 individual plants during the middle of the light period, immediately frozen in liquid nitrogen, and stored at—80°C until use.

### Light Microscopy

Samples of fully expanded cotyledons and assimilating shoots were fixed in Formalin–acetic acid–alcohol (FAA) for 24 hours, dehydrated through an alcohol series, cleared with xylene, and embedded in paraffin wax. Cross-sections were obtained using a microtome. For light microscopy, semi-thin sections were stained with safranin O solution and studied under DIC microscope (BX51, Olympus, Japan) equipped with an LM Digital Camera (DP70, Olympus).

### Stable Carbon Isotope Analysis

Stable carbon isotope ratios (^13^C/^12^C) were quantified in cotyledons and dried assimilating shoots from plants grown in the greenhouse. Then, 1–2 cm segments of the middle regions of fully expanded cotyledons or assimilating shoots were collected. All samples were oven-dried at 65°C for 48 h to a constant weight.

The measurements of stable carbon isotope ratios were carried out at the Chinese Academy of Forestry’s Stable Isotope Laboratory (Beijing, China) using a Flash EA1112 HT elemental analyzer (Thermo Scientific) coupled with a Delta V advantage isotope ratio mass spectrometer (Thermo Scientific). Stable carbon isotope ratios were expressed as δ^13^C (‰), calculated as follows:

$${\text{δ}}^{\text{13}}\text{C }\left(\text{‰}\right)\text{ = }\left[\left({\text{R}}_{\text{sample}}{\text{/ R}}_{\text{standard}}\right)\text{-1}\right]\text{×1000}$$

where R_sample_ and R_standard_ are the ^13^C/^12^C ratios for an individual sample and the reference standard (Pee Dee Belemnite), respectively.

### RNA Preparation and Sequencing

Total RNA was prepared with TRIzol according to the manufacturer’s instructions (Invitrogen Life Technologies, Shanghai, China). Following extraction, total RNA was purified using the RNeasy Mini Kit from Qiagen (Shanghai, China), including on-column DNase digestion (Qiagen, Shanghai, China). Purified RNA was checked for integrity and quality using an Agilent 2100 Bioanalyzer (Agilent Technologies, Santa Clara, CA, USA). The cDNA library was constructed for sequencing as described in the Illumina TruSeq RNA sample preparation v2 guide (Catalog # RS-930–1021). Sequencing was performed on an Illumina HiSeq 2000.

### Mapping and Quantification of the Sequence Reads

We filtered and examined the quality of the raw sequence reads as described by Xu et al. [37]. The first 10 bases in each read were trimmed off before the mapping process.

**Mapping and Quantification of the Sequence Reads from Cotyledons and Leaves of *Arabidopsis***. Clean reads were mapped onto the latest *Arabidopsis thaliana* genome assembly (http://www.phytozome.net/arabidopsis.php) using the Bowtie2 (http://bowtie-bio.sourceforge.net/bowtie2/index.shtml) [38]. The best hit of each read with a maximum of three nucleotide mismatches was used. For each Arabidopsis Genome Initiative (AGI) code, the number of matching reads was counted, and the raw digital gene expression counts were normalized using the RPKM (Reads Per Kilobase per Million mapped reads) method [39,40].

**Mapping and Quantification of the Sequence Reads from the Cotyledons and Assimilating Shoots of *H*. *ammodendron***. Clean reads were mapped onto the coding sequences of the latest *A*. *thaliana* genome assembly (http://www.phytozome.net/arabidopsis.php) using BLAT [41]. Alignments were performed in the protein space, and the best hit for each read was retained. For each Arabidopsis Genome Initiative (AGI) code, the number of matching reads was counted, and the raw digital gene expression counts were normalized using the RPKM method [39,40].

The differential expression between samples was statistically accessed by the R/Bioconductor package edgeR [42]. Genes with FDR≤0.001 and |log_2_(Fold change)|≥1 were considered significant.

### Overrepresentation Analysis

To identify functional categories with significant differences between cotyledons and assimilating shoots, we performed an overrepresentation analysis using the GO Term Enrichment of AmiGO (http://amigo.geneontology.org/amigo) [43]. We used all detected transcripts in cotyledons and assimilating shoots as the background set and TAIR as the filter.

## Results

### Photosynthetic Features of Assimilating Shoots and Cotyledons

**Figure 1:**
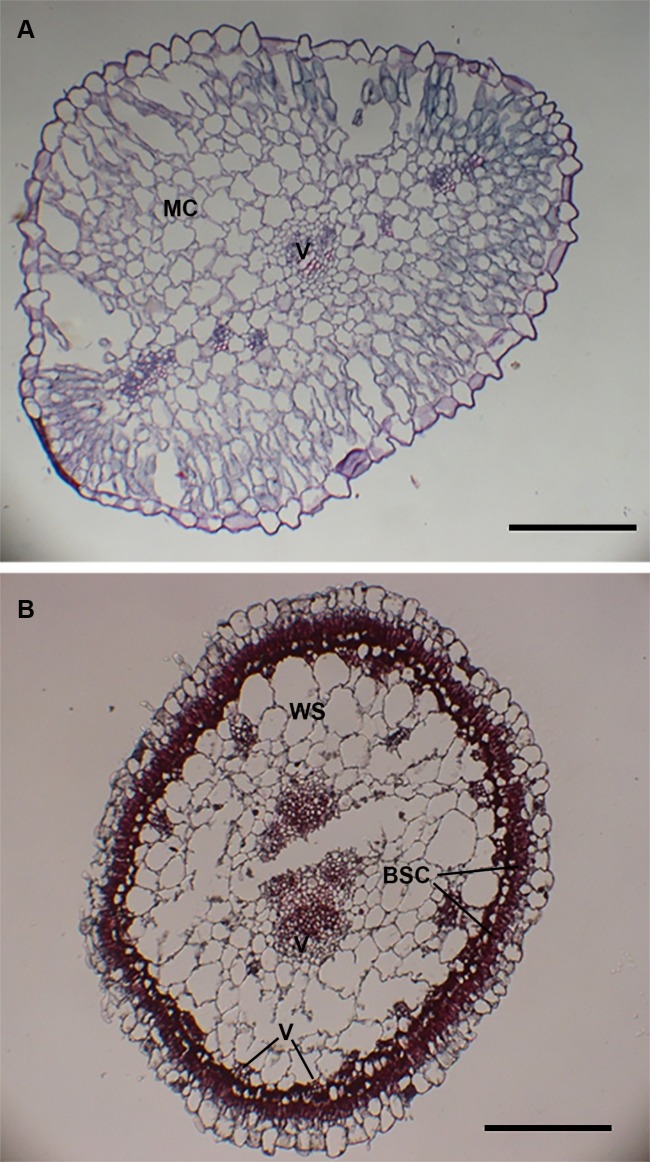
Transverse sections of a cotyledon (A) and an assimilating shoot (B) of *Haloxylon ammodendron*. MC, mesophyll cell; BSC, bundle sheath cell; WS, water storage tissue; V, vascular tissue. Scale bars represent 100 μm.

*Haloxylon* species have an unusual photosynthetic apparatus. The true leaves are reduced, and the young annual cylindrical shoots (assimilating shoots) are the main photosynthetic tissues. Fifteen years ago, Pyankov et al. [29] discovered that two *Haloxylon* species, *H*. *aphyllum* and *H*. *persicum*, have a C_4_ type of photosynthesis in assimilating shoots with Kranz anatomy, whereas leaf-like cotyledons lack Kranz-anatomy and incorporate CO_2_ via C_3_ photosynthesis, as observed through analyses of stable carbon isotope ratios, anatomy, primary photosynthetic products, and activities of carbon metabolism enzymes. We examined the anatomy assimilating shoots and cotyledons in *H*. *ammodendron* and confirmed that *H*. *ammodendron*, similarly to the other two *Haloxylon* species, uses different types of photosynthesis in assimilating shoots and cotyledons, as shown in Fig. 1. The cotyledons have no Kranz-type anatomy and several layers of mesophyll cells around only a few vascular bundles (Fig. 1A). The assimilating shoots have Salsoloid-type Kranz anatomy with two continuous layers of chlorenchyma (a layer of palisade mesophyll cells and an inner layer of bundle sheath cells) on the periphery and water-storage parenchyma in the center (Fig. 1B). The main vascular bundle occupies the central position, and only the small, peripheral vascular bundles are in contact with bundle sheath cells (Fig. 1B).

Stable carbon isotope ratios are used to distinguish the photosynthetic CO_2_-fixing pathways of plants [44–46]. To further confirm the photosynthetic types of cotyledons and assimilating shoots, we analyzed the stable carbon isotope ratios by measuring δ^13^C values. The δ^13^C value for *H*. *ammodendron* cotyledons was-15.58±0.72 ‰ (mean ± SE, n = 3), similar to that of *H*. *aphyllum* and *H*. *persicum* reported by Pyankov et al. (-17.5 ‰) [29]. Unexpectedly, the assimilating shoots exhibited a more negative δ^13^C value of-21.89±1.05 ‰ (mean ± SE, n = 3).

### Major Transcriptional Changes

To identify differences in transcript abundance related to C_4_ syndrome, the transcriptomes of *H*. *ammodendron* assimilating shoots and cotyledons were compared. cDNA libraries of assimilating shoots and cotyledons were constructed and sequenced using the Illumina HiSeq 2000 platform, resulting in 30,287,044 and 36,971,687 reads, respectively, with a mean read length of 101 nucleotides. After trimming adapters and filtering out low-quality reads, 29,558,368 reads from assimilating shoots and 36,070,605 reads from cotyledons were retained for further analysis. Clean reads were mapped onto the coding sequences of the latest A. thaliana genome assembly (http://www.phytozome.net/arabidopsis.php) using BLAT [41]. 27037741 reads (~75.0%) from cotyledons and 23188916 reads (~78.5%) from assimilating shoots could be mapped onto the *Arabidopsis* transcriptome.

mRNA-Seq analysis comparing the transcriptomes of *H*. *ammodendron* assimilating shoots and cotyledons yielded 2959 differentially expressed genes [FDR≤0.001 and abs (|log_2_(Fold change)|≥1)], with 1852 and 1107 more abundant transcripts in assimilating shoots and cotyledons, respectively (see Table A in S1 File). To test whether these differentially expressed transcripts are enriched in functional categories, we performed overrepresentation analysis using the GO Term Enrichment of AmiGO. The significantly up-regulated transcripts in assimilating shoots were enriched in several fundamental biological process categories, including methylation, cytokinesis, DNA replication, cell wall organization or biogenesis, biosynthetic process, anatomical structure morphogenesis, signaling pathway and developmental process (see Table B in S1 File). Down-regulated GO categories are less abundant than up-regulated ones, mainly in response to endogenous stimulus (see Table C in S1 File). Because we used TAIR as a filter and *Arabidopsis* is a typical C_3_ plant, no functional C_4_ class was detected.

### C_4_ Cycle Genes Were Up-regulated in Assimilating Shoots

Transcript analysis of known C_4_ genes showed that all genes necessary for the core C_4_ cycle of NADP-ME type plants were significantly up-regulated in assimilating shoots compared with cotyledons (Table 1). Among these genes, NADP-ME was most significantly different, with a 12.9- (At5g11670), 12.7- (At2g19900) or 9.3- (At1g79750) fold higher transcript abundance in assimilating shoots. The second biggest difference came from PEPC, with a 12.1- (At2g42600) or 10.5- (At1g53310) fold up-regulation. Additionally, transcripts encoding AspAT (At4g31990) and the chloroplastidic MDH (At5g58330) were up-regulated 8.5- and 2.1-fold, respectively. Transcripts encoding PPDK (At4g15530) were increased 2.7-fold.

**Table 1:** Transcript abundance of C_4_ cycle genes and C_4_-related transporters.

Gene ID	Protein	Ha-AS(RPKM)	Ha-C(RPKM)	Fold Change	log_2_(Ha-AS/Ha-C)
At4g15530	PPDK	5768.71	2133.50	2.70	1.44
At1g53310	PEPC	1081.51	102.90	10.51	3.39
At2g42600	PEPC	1201.30	99.31	12.10	3.60
At1g68750	PEPC	2.84	6.96	0.41	-1.29
At5g58330	cpNADP-MDH	1287.07	601.46	2.14	1.10
At1g79750	NADP-ME	814.71	87.96	9.26	3.21
At2g19900	NADP-ME	2480.24	194.89	12.73	3.67
At5g11670	NADP-ME	1161.41	90.10	12.89	3.69
At1g17290	AlaAT	393.42	103.14	3.81	1.93
At4g31990	AspAT	748.92	87.97	8.51	3.09
At5g19550	AspAT	59.18	80.65	0.73	-0.45
At5g11520	AspAT	79.59	112.71	0.71	-0.50
At2g22250	AspAT	37.61	28.50	1.32	0.40
At1g53240	mtNAD-MDH	190.23	129.15	1.47	0.56
At4g00570	NAD-ME	42.94	27.82	1.54	0.63
At4g37870	PEP-CK	125.01	168.60	0.74	-0.43
At3g47520	cpNAD-MDH	90.47	34.47	2.62	1.39
At1g08650	PEPC-K	17.44	1.59	10.98	3.46
At5g47840	AMK2	489.96	230.02	2.13	1.09
At5g35170	adenylate kinase family protein	141.21	216.67	0.65	-0.62
At5g09650	PPA6	1042.96	739.28	1.41	0.50
At2g26900	BASS 2	1083.92	249.28	4.35	2.12
At3g56160	BASS 4	39.89	6.88	5.80	2.54
At5g33320	PPT1	225.80	39.88	5.66	2.50
At3g01550	PPT2	25.57	4.82	5.30	2.41
At5g46110	TPT	1386.73	2111.57	0.66	-0.61
At5g12860	Dit1	390.82	259.62	1.51	0.59
At5g64280	Dit2	91.71	24.54	3.74	1.90

Ha-AS = *Haloxylon ammodendron* assimilating shoots, Ha-C = *Haloxylon ammodendron* cotyledons.

All genes required for the NAD-ME type of C_4_ photosynthesis were also up-regulated in assimilating shoots compared with cotyledons (Table 1). The transcripts encoding NAD-ME (At4g00570) were up-regulated 1.5-fold, and those encoding AlaAT (At1g17290) and mtNAD-MDH (At1g53240) were up-regulated 3.8- and 1.5-fold, respectively.

Transcripts encoding the regulatory factors PEPC kinase (PEPC-K) increased 11-fold, which was significant (Table 1). Additionally, the transport proteins required for C_4_ photosynthesis were significantly up-regulated (see Table 1), such as pyruvate sodium symporter (BASS2), phosphoenolpyruvate/phosphate translocators (PPT1 and PPT2) and chloroplast dicarboxylate transporters (DiT1 and DiT2).

### Photorespiratory Genes Were Down-regulated in Assimilating Shoots

A major advantage of the C_4_ pathway is the reduction in photorespiration because high CO_2_ concentration around Rubisco in bundle sheath cells effectively suppresses photorespiration. Detailed analysis of gene expression of all photorespiration genes showed that the transcripts of nearly all genes related to photorespiration were lower in abundance in assimilating shoots than in cotyledons (Table 2). The genes AtAGT1 (At2g13360), AtGGT1 (At1g23310), AtGLDT1 (At1g11860), AtSHM1 (At4g37930) and AtGLYK (At1g80380), all of which play major roles in photorespiration, were significantly down-regulated (log_2_ (Fold change) ≤-1).

**Table 2:** Transcript abundance of photorespiration genes.

Enzyme	Gene name	Gene ID	Ha-AS(RPKM)	Ha-C(RPKM)	log_2_(Ha-AS/Ha-C)
2PG phosphatase	AtPGLP1	At5g36790*	243.47	228.19	0.09
Glycolate oxidase	AtGOX1	At3g14420	911.87	3690.36	-2.02
	AtGOX2	At3g14415	330.80	1326.77	-2.00
	AtGOX3	At4g18360	151.29	679.40	-2.17
	AtHAOX1	At3g14130	8.32	12.51	-0.59
	AtHAOX2	At3g14150	9.20	16.56	-0.85
Ser:glyoxylate aminotransferase	AtAGT1	At2g13360	362.28	2052.76	-2.50
Glu:glyoxylate aminotransferase	AtGGT1	At1g23310	670.03	1607.05	-1.26
	AtGGT2	At1g70580	527.48	1194.60	-1.18
Gly decarboxylase P-protein	AtGLDP1	At4g33010	503.01	1461.69	-1.54
	AtGLDP2	At2g26080	266.08	770.75	-1.53
Gly decarboxylase H-protein	AtGLDH1	At2g35370	153.54	644.93	-2.07
	AtGLDH2	At2g35120	84.97	54.76	0.63
	AtGLDH3	At1g32470	168.58	721.63	-2.10
Gly decarboxylase T-protein	AtGLDT1	At1g11860	546.86	1701.11	-1.64
Gly decarboxylase L-protein	AtmLPD1	At3g17240	164.91	271.07	-0.72
	AtmLPD2	At1g48030	156.18	277.42	-0.83
Ser hydroxymethyltransferase	AtSHM1	At4g37930	696.67	1457.01	-1.06
	AtSHM2	At5g26780	378.13	774.57	-1.03
Hydroxypyruvate reductases	AtHPR1	At1g68010	647.42	1269.02	-0.97
	AtHPR2	At1g79870*	115.77	104.57	0.15
Glycerate kinase	AtGLYK	At1g80380	46.14	115.70	-1.33

Ha-AS = *Haloxylon ammodendron* assimilating shoots, Ha-C = *Haloxylon ammodendron* cotyledons.

*no significant differences in the expression of these genes between cotyledons and assimilating shoots (p>0.01).

### Genes Controlling Vein Density Were Up-regulated in Assimilating Shoots

Kranz anatomy is accompanied by high vein density [16], so we assessed the transcript abundance of known genes controlling vein density. All of these genes were up-regulated in assimilating shoots (Table 3). Among them, the transcription factors MONOPTEROS/AUXIN RESPONSE FACTOR5 (MP/ARF5) MP and ATHB8, which are involved in the auxin signal transduction pathway controlling leaf vascular development, were up-regulated 3.9- and 1.9-fold, respectively.

**Table 3:** Transcription abundance of known genes related to vein density.

Gene ID	Name	Ha-AS(RPKM)	Ha-C (RPKM)	Fold Change	log_2_(Ha-AS/Ha-C)
At4g32880	ATHB8	13.79	3.53	3.91	1.97
At1g19850	MP/ARF5	6.05	3.26	1.86	0.89
At1g13980	GNOM/EMB30	22.25	18.68	1.19	0.25
At2g36120	DOT1	248.82	157.52	1.58	0.66
At1g13290	DOT5	3.16	1.86	1.70	0.77
At5g60690	REVOLUTA (REV)	15.66	5.91	2.65	1.41
At5g13300	SFC	14.41	9.46	1.52	0.61
At1g73590	PIN-FORMED1 (PIN1)	10.80	4.00	2.70	1.43
At1g20330	CVP1/SMT2	37.70	20.13	1.87	0.91
At1g05470	CVP2	2.95	1.28	2.31	1.20
At5g55540	LOP1(now tornado1–2)	1.09	0.19	5.72	2.52
At1g65620	AS2	0.53	0.00		19.02
At5g60200	DOF5.3	1.94	0.92	2.10	1.07

Ha-AS = *Haloxylon ammodendron* assimilating shoots, Ha-C = *Haloxylon ammodendron* cotyledons.

### Differentially Expressed Transcription Factors Encoding Genes

A total of 248 transcription factor-encoding genes were identified, showing differential expression [FDR≤0.001 and abs (|log_2_(Fold change)|≥1)] in assimilating shoots compared with cotyledons (Table 4). In total, 107 genes were up-regulated, and 141 genes were repressed in assimilating shoots. To avoid capturing variation in transcript abundance associated with differences between the different developmental stages that do not relate to C_4_ photosynthesis, we also sequenced the transcriptomes of *Arabidopsis* cotyledons and leaves in parallel to minimize the influence of developmental stage-specific effects. Excluding the genes that had the same developmental expression pattern in *H*. *ammodendron* and *Arabidopsis*, there were 96 putative positive regulators and 130 putative negative regulators. We tested the likelihood that SCARECROW/SHORTROOT components [47] of the C_4_ regulatory network were among these identified genes (see Table 5). Scarecrow plays a role in establishing Kranz anatomy in maize leaves, and its mutation results in abnormal Kranz anatomy [48]. SHR was included among the identified genes, and SCR was up-regulated by 1.3-fold in assimilating shoots, although it was not identified.

**Table 4:** Differentially expressed transcription factor-encoding genes.

Putative positive regulators					
At1g01250	AP2-EREBP family	At3g57670	C2H2 family	At2g34710	Homeobox family
At1g68550*	AP2-EREBP family	At4g27240	C2H2 family	At3g18010	Homeobox family
At1g79700	AP2-EREBP family	At5g03740*	C2H2 family	At4g08150	Homeobox family
At4g16750	AP2-EREBP family	At5g39550	C2H2 family	At4g32880	Homeobox family
At4g23750*	AP2-EREBP family	At5g54630	C2H2 family	At5g46880	Homeobox family
At4g37750	AP2-EREBP family	At5g57520	C2H2 family	At5g60690	Homeobox family
At5g11190	AP2-EREBP family	At1g68200	C3H family	At1g24260	MADS family
At5g17430	AP2-EREBP family	At2g44580	C3H family	At2g03710	MADS family
At5g57390	AP2-EREBP family	At3g63530	C3H family	At2g45660	MADS family
At2g46530	ARF family	At5g45290	C3H family	At3g30260	MADS family
At1g27660	bHLH family	At1g54160	CCAAT-HAP2 family	At4g22950	MADS family
At1g61660	bHLH family	At3g14020*	CCAAT-HAP2 family	At4g24540*	MADS family
At1g68810	bHLH family	At5g12840	CCAAT-HAP2 family	At5g15800	MADS family
At2g27230	bHLH family	At5g06510*	CCAAT-HAP2 family	At5g60910	MADS family
At2g41130	bHLH family	At1g08970	CCAAT-HAP5 family	At2g47460	MYB family
At3g06120	bHLH family	At5g43250	CCAAT-HAP5 family	At3g61250*	MYB family
At3g20640	bHLH family	At3g04850	CPP family	At4g32730	MYB family
At5g10570	bHLH family	At3g22760	CPP family	At5g15310	MYB family
At5g57150	bHLH family	At3g22780	CPP family	At5g49330	MYB family
At5g65640	bHLH family	At4g14770	CPP family	At1g54330	NAC family
At2g18160	bZIP family	At3g01330	E2F-DP family	At1g62700	NAC family
At1g28050	C2C2-CO-like family	At3g48160	E2F-DP family	At3g57150	NAC family
At2g33500	C2C3-CO-like family	At3g10760	G2-like	At4g28500	NAC family
At3g50410	C2C2-Dof family	At3g46640	G2-like	At2g24630	REM family
At1g08000	C2C2-Gata family	At5g42630	G2-like	At1g02065	SBP family
At3g06740	C2C3-Gata family	At1g50420*	GRAS family	At1g69170	SBP family
At4g16141	C2C4-Gata family	At1g63100	GRAS family	At5g43270	SBP family
At5g26930	C2C5-Gata family	At4g37650	GRAS family	At4g37490	TCP family
At5g49300	C2C6-Gata family	At5g41920	GRAS family	At1g16070	TUB family
At2g45190	C2C2-YABBY family	At2g22840	GRF family	At4g39410	WRKY family
At4g00180	C2C2-YABBY family	At1g30490	Homeobox family	At1g75240	ZF-HD family
At1g75710	C2H2 family	At1g46480	Homeobox family	At2g02540	ZF-HD family
At2g29660	C2H2 family	At1g52150*	Homeobox family	At2g18350	ZF-HD family
At3g12270	C2H2 family	At1g62990	Homeobox family	At4g24660	ZF-HD family
At3g14740	C2H2 family	At1g79840	Homeobox family	At5g65410	ZF-HD family
At3g44750*	C2H2 family	At2g27990	Homeobox family		
Putative negative regulators					
At1g01030	ABI3VP1 family	At5g50915	bHLH family	At2g46680	Homeobox family
At1g25560	ABI3VP1 family	At5g62610	bHLH family	At3g01220	Homeobox family
At1g03800	AP2-EREBP family	At5g67060	bHLH family	At3g61890	Homeobox family
At1g12610*	AP2-EREBP family	At1g08320	bZIP family	At5g15150	Homeobox family
At1g19210	AP2-EREBP family	At3g10800	bZIP family	At5g47370	Homeobox family
At1g46768	AP2-EREBP family	At1g25440	C2C2-CO-like family	At4g26170	HRT family
At1g50640	AP2-EREBP family	At1g49130	C2C2-CO-like family	At4g36990	HSF family
At1g64380	AP2-EREBP family	At1g68520	C2C2-CO-like family	At5g62020	HSF family
At1g74930	AP2-EREBP family	At1g73870	C2C2-CO-like family	At1g68320	MYB family
At2g28550	AP2-EREBP family	At2g24790	C2C2-CO-like family	At1g52890	NAC family
At2g40340	AP2-EREBP family	At3g02380	C2C2-CO-like family	At1g01720	NAC family
At3g11020	AP2-EREBP family	At4g39070	C2C2-CO-like family	At1g52880	NAC family
At3g15210	AP2-EREBP family	At5g15840	C2C2-CO-like family	At1g69490	NAC family
At3g20310	AP2-EREBP family	At5g15850	C2C2-CO-like family	At1g77450	NAC family
At3g54990*	AP2-EREBP family	At5g57660	C2C2-CO-like family	At2g17040	NAC family
At4g17490	AP2-EREBP family	At1g29160	C2C2-Dof family	At4g17980	NAC family
At4g25470	AP2-EREBP family	At1g51700	C2C2-Dof family	At4g27410	NAC family
At4g25490	AP2-EREBP family	At1g64620	C2C2-Dof family	At4g28530	NAC family
At4g34410*	AP2-EREBP family	At1g69570	C2C2-Dof family	At5g08790	NAC family
At4g36900	AP2-EREBP family	At3g21270	C2C2-Dof family	At5g18270	NAC family
At5g05410	AP2-EREBP family	At1g27730	C2H2 family	At5g39610*	NAC family
At5g07310	AP2-EREBP family	At3g19580	C2H2 family	At5g61430	NAC family
At5g13330	AP2-EREBP family	At3g49930	C2H2 family	At5g63790	NAC family
At5g25190*	AP2-EREBP family	At5g12850	C2H2 family	At1g64530	NLP family
At5g44210	AP2-EREBP family	At5g04340	C2H2 family	At1g13260	RAV family
At5g47230	AP2-EREBP family	At5g67450	C2H2 family	At1g68840*	RAV family
At5g50080	AP2-EREBP family	At1g26800	C3H family	At2g36080	RAV family
At5g51190	AP2-EREBP family	At2g15580	C3H family	At2g46870*	RAV family
At5g51990	AP2-EREBP family	At3g10910	C3H family	At3g11580	RAV family
At5g61600	AP2-EREBP family	At3g58720	C3H family	At3g25730	RAV family
At5g61890	AP2-EREBP family	At4g13100	C3H family	At5g06250	RAV family
At5g64750*	AP2-EREBP family	At1g67910	CAMTA family	At1g53230	TCP family
At5g20730	ARF family	At2g13570	CCAAT-HAP3 family	At2g38250	Trihelix family
At1g76110	ARID family	At1g68670	G2-like family	At5g01380	Trihelix family
At1g04880	ARID family	At2g03500	G2-like family	At1g29860	WRKY family
At3g48100	ARR-B family	At3g04030	G2-like family	At1g62300	WRKY family
At1g09530	bHLH family	At5g18240	G2-like family	At1g69310	WRKY family
At1g18400	bHLH family	At1g07520	GRAS family	At1g80840*	WRKY family
At1g22380	bHLH family	At1g07530	GRAS family	At2g23320	WRKY family
At1g26260	bHLH family	At2g29060	GRAS family	At2g38470	WRKY family
At1g73830*	bHLH family	At2g37650	GRAS family	At2g47260	WRKY family
At2g18300	bHLH family	At4g17230	GRAS family	At4g04450	WRKY family
At2g20180	bHLH family	At4g24150	GRF family	At4g18170	WRKY family
At3g07340	bHLH family	At5g53660	GRF family	At4g22070	WRKY family
At3g21330	bHLH family	At1g26960	Homeobox family	At4g31800	WRKY family
At4g34530*	bHLH family	At1g69780	Homeobox family	At5g26170	WRKY family
At4g36540	bHLH family	At2g44910	Homeobox family	At5g46350*	WRKY family

Ha-AS = *Haloxylon ammodendron* assimilating shoots, Ha-C = *Haloxylon ammodendron* cotyledons.

*these genes had the same developmental expression pattern in *H*. *ammodendron* and *Arabidopsis*.

**Table 5:** SCARECROW/SHORTROOT regulatory network.

Maize Gene ID	Arabidopsis Gene ID	Arabidopsis ortholog	Hal-AS (RPKM)	Hal-C (RPKM)	log_2_(Ha-AS/Ha-C)
GRMZM2G131516	AT3G54220	SCR	27.26	20.31	0.42
GRMZM2G132794	AT4G37650	SHR	12.63	0.90	3.81
GRMZM2G172657					
GRMZM2G150011	AT1G13290	DOT5	3.16	1.86	0.77

Ha-AS = *Haloxylon ammodendron* assimilating shoots, Ha-C = *Haloxylon ammodendron* cotyledons.

## Discussion

The *Haloxylon* genus comprises three closely related species, including *H*. *ammodendron* (saxaul), *H*. *aphyllum* (black saxaul) and *H*. *persicum* (white saxaul). *H*. *aphyllum and H*. *persicum* were reported to have different types of photosynthesis in assimilating shoots and cotyledons. Similarly, *H*. *ammodendron* assimilating shoots had Salsoloid-type Kranz anatomy (Fig. 1B), indicative of C_4_ photosynthesis, while the structure of cotyledons was of the non-Kranz type (Fig. 1A). The developmental transition from a C_3_ pathway to a two-celled C_4_ pathway in *Haloxylon* species in nature is a good system with which to study the genetic regulatory network of C_4_ syndrome.

Stable carbon isotope analysis is used as a screening method to determine the photosynthetic pathway when it is unknown in a species [46]. We measured the δ^13^C values of cotyledons and assimilating shoots. The *H*. *ammodendron* cotyledons had a δ^13^C value of-15.58±0.72 ‰ (mean ± SE, n = 3), falling into the range of typical values for C_4_ plants of-6 to-19 ‰ [49]. Although this finding is contrary to the anatomical results, it is consistent with a previous report of *Haloxylon* that proposed that these C_4_-type values are a result of old C_4_ assimilates stored in the cotyledons during seed formation [29]. Interestingly, the assimilating shoots exhibited a δ^13^C value of-21.89±1.05 ‰ (mean ± SE, n = 3) and were closer to that of C_3_ plants (-23 ‰ to-32 ‰). Because *Haloxylon* seeds have no endosperm, cotyledon photosynthesis provides C_3_ assimilates to support early plant development [29]. *H*. *ammodendron* has two large and long-lived cotyledons; therefore, the δ^13^C values of young assimilating shoots (10 days of age) are more negative and closer to that of C_3_ due to a mixture of assimilates from C_3_ (cotyledons) and C_4_ photosynthesis (assimilating shoots).

In this study, we performed deep mRNA-Seq using the Illumina HiSeq 2000 platform to analyze the transcriptomes of *H*. *ammodendron* assimilating shoots and cotyledons, which exhibit different types of photosynthesis. Using *Arabidopsis* as the reference genome, 2959 differentially expressed genes [FDR≤0.001 and abs (|log_2_(Fold change)|≥1)] were identified, with 1852 and 1107 transcripts being more abundant in assimilating shoots and cotyledons, respectively (see Table A in S1 File).

It was reported that the assimilating shoots of *H*. *aphyllum and H*. *persicum* mainly perform NADP-ME-type C_4_ shuttling and a small fraction of NAD-ME-type C_4_ shuttling, as shown by photosynthetic enzyme activity analysis and immunoblot analysis [29]. The mRNA-Seq analysis presented here confirmed this discovery and further showed that up-regulation occurs at the level of transcript abundance. All genes necessary for the core C_4_ cycle of NADP-ME type plants were significantly up-regulated, and all genes required for the NAD-ME type of C_4_ photosynthesis were also up-regulated, but to a lesser extent, in assimilating shoots compared with cotyledons (Table 1). This suggests that NADP-ME-type C_4_ photosynthesis is predominant, and NAD-ME-type C_4_ photosynthesis makes a small contribution to photosynthetic CO_2_ fixation. Likewise, nearly all genes encoding photorespiratory proteins had lower steady-state transcriptional levels in assimilating shoots (Table 2).

We identified a list of positive and negative regulators of C_4_ syndrome (Table 4). Although not all of these transcription factors are known to be components of the C_4_ regulatory network, our observations suggest that at least a subset of these factors are very likely involved in vein density and the development of Kranz anatomy. Within the list, the transcription factor ATHB8 (Table 4) and the upstream gene MP (Table 3) in the auxin signal transduction pathway controlling leaf vascular development were both up-regulated. This evidence is supportive of a role for at least a subset cohort in vein density. Very recently, Wang et al. (2013) performed a genome-wide comparative analysis of developmental trajectories in Kranz (foliar leaf blade) and non-Kranz (husk leaf sheath) leaves of the C_4_ plant maize to look for regulators of Kranz anatomy and identified 48 putative positive regulators, of which, 40 genes were assigned to 38 *Arabidopsis* orthologous genes [47]. Although maize has a Panicoid-type (classical NADP-ME type) Kranz anatomy and *Haloxylon* species have a Salsoloid-type Kranz anatomy, there remains high overlap between the list of positive regulators in each study. Within our list of putative positive regulators of C_4_ syndrome, 11 of 38 *Arabidopsis* transcription factor-encoding genes were also significantly up-regulated in *H*. *ammodendron* assimilating shoots. Another two genes, AT3G54220 and AT3G13960, were increased by 1.3- and 1.9-fold (Table 6). This high overlap ratio confirms that the methods we used identified Kranz anatomy regulators.

**Table 6:** High overlap between putative positive Kranz regulators identified by Wang et al. (2013) and this study.

Maize Gene ID	Annotation	Arabidopsis Gene ID	Arabidopsis ortholog	Ha-AS (RPKM)	Ha-C (RPKM)	log_2_(Ha-AS/Ha-C)
GRMZM2G132794	GRAS (SHR)	AT4G37650	SHR	12.63	0.90	3.81
GRMZM2G172657	GRAS (SHR)					
GRMZM2G163975	bHLH family	AT1G27660		5.00	2.43	1.04
GRMZM2G039074	Myb family	AT5G42630	ATS	1.04	0.06	4.03
GRMZM2G178182	bHLH family	AT5G50915		7.44	15.34	-1.04
GRMZM2G472945	TLP-family	AT1G16070	TLP8	1.68	0.17	3.34
GRMZM2G178102	HD-ZIP III (PHV-like)	AT1G30490	PHV	2.63	0.90	1.55
GRMZM2G131516	GRAS (SCR)	AT3G54220	SCR	27.26	20.31	0.42
GRMZM2G136494	MRPI-like ZnF	AT1G75710	ANT	13.51	4.30	1.65
GRMZM2G028046	MRPI-like ZnF					
GRMZM2G021573	AP2-EREBP (ANT-like)	AT4G37750		7.82	0.93	3.07
GRMZM2G146688	AP2-EREBP (ANT-like)					
GRMZM2G040924	MIXTA-like Myb	AT3G61250		1.95	0.51	1.95
GRMZM2G111045	MIXTA-like Myb					
GRMZM2G171365	ZmMADS1	AT2G45660	AGL20	19.94	0.93	4.43
GRMZM2G425236	ZnF-HD family	AT4G24660	ATHB22	28.69	7.66	1.91
GRMZM2G097275	SBP family	AT5G43270	SPL2	0.77	0.21	1.91
GRMZM5G850129	GRF	AT3G13960	GRF5	6.71	3.50	0.94

Ha-AS = *Haloxylon ammodendron* assimilating shoots, Ha-C = *Haloxylon ammodendron* cotyledons. Wang et al. (2013) identified 48 putative positive Kranz regulators through comparative analysis of Kranz (foliar leaf blade) and non-Kranz (husk leaf sheath) leaves of maize. Forty genes were assigned to 38 *Arabidopsis* orthologous genes [47]. Fourteen of 38 *Arabidopsis* transcription factor-encoding genes were also identified in this study, and 11 transcription factors were significantly up-regulated (log_2_ (Fold change) >1) in *H*. *ammodendron* assimilating shoots.

The differences in transcript abundance between the different photosynthetic organs of *H*. *ammodendron*, cotyledons and assimilating shoots, reflect the development of C_4_ syndrome. Previous studies suggested C_4_ cycle genes, trans-factors and even *cis*-elements were recruited from ancestral C_3_ plants [50–53], but the mechanism behind is not clear. With the same genomic context of the cotyledons and assimilating shoots, this natural and developmental transition from C_3_ to C_4_ would be an ideal model system to study the molecular mechanism of recruitment, if whole genome sequence and genetic transformation are available.

## Supporting Information

S1 Fig *Haloxylon ammodendron* grown in its natural habitat. (TIF)

S1 File Contains Tables A-C. Table A. 2959 differentially expressed genes [FDR≤0.001 and abs (|log2(Fold change)|≥1)] Table B. Up-regulated GO categories. Table C. Down-regulated GO categories.
(XLSX)
