# Supplementary figures and images for: Developmental Genetic Mechanisms of C4 Syndrome Based on Transcriptome Analysis of C3 Cotyledons and C4 Assimilating Shoots in Haloxylon ammodendron
Source: PLoS One. 2015 Feb 2;10(2):e0117175. doi: 10.1371/journal.pone.0117175 (PMC4313948; doi:10.1371/journal.pone.0117175)

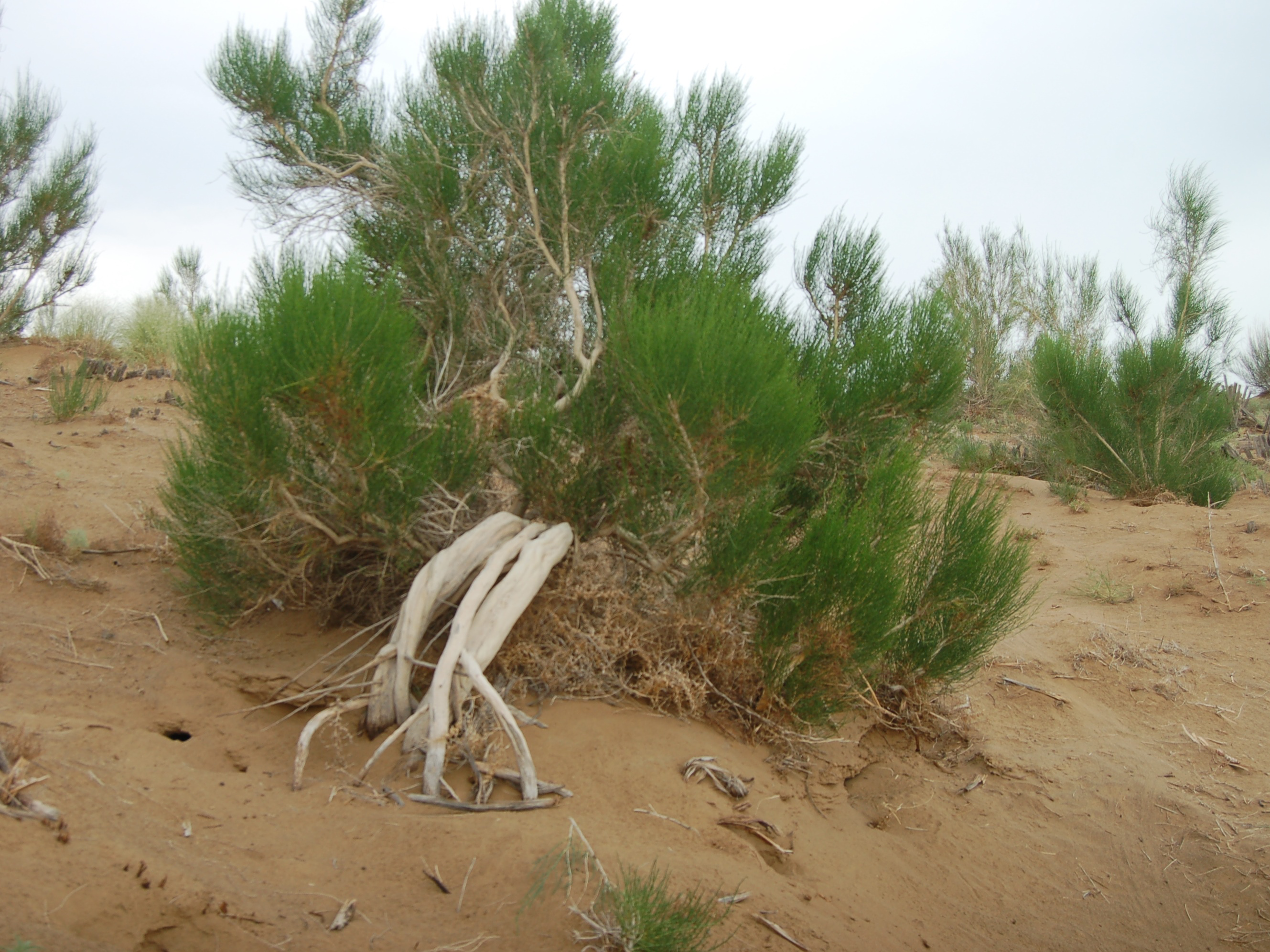

Supplement: S1 Fig — (TIF) [file pone.0117175.s001.tif]
